# A new financial settlement approach to stabilize profitability of pig production

**DOI:** 10.1371/journal.pone.0304949

**Published:** 2024-06-10

**Authors:** Michał Litwiński, Paulina Luiza Wiza-Augustyniak, Łukasz Kryszak, Wojciech Styburski

**Affiliations:** 1 Department of Sociology and Business Ethics, Poznan University of Economics and Business, Poznan, Poland; 2 Faculty of Sociology, Adam Mickiewicz University in Poznan, Poznan, Poland; 3 Chair of Law and Organisation of Enterprises in Agribusiness, Poznań University of Life Sciences, Poznan, Poland; 4 Department of Macroeconomics and Agricultural Economics, Poznan University of Economics and Business, Poznan, Poland; 5 Department of Economics and Economic Policy in Agribusiness, Poznań University of Life Sciences, Poznan, Poland; University of Life Sciences in Lublin, POLAND

## Abstract

This article proposes and evaluates a new solution that ensures the profitability in short and medium terms and stability of the operations of pork livestock producers through improved risk management An innovative tool for distributing the surplus between producers of piglets and finishers is presented. Manuals on pig farming and data combined from multiple sources were used to assess the current market situation, design a profit stabilization tool for pig producers, and evaluate the performance of this solution. We found that implementing the tool reduces the profits variability of finishers and piglets producers by 45% and 30%, respectively, while keeping the long-term average of profits constant.

## Introduction

Economic fluctuations have a powerful impact on the performance and stability of agricultural activities. Assessing the impact of the economic climate is particularly important for pig production. Due to price fluctuations and a strong correlation between the prices of piglets and finishers (the “ups” and “downs” of pig prices), it is quite common for a producer of finishers to suffer a loss. A typical fattening period, during which 30-kg piglets become 130-kg finishers, is 14 weeks. However, farmers cannot predict, when they are buying piglets, what the price of finishers will be at the end of the fattening period. As a result, there is a high likelihood that piglets will be purchased at the “up” (high prices), and the sale of the finisher will take place at the “down” (low prices). In other words, by buying an expensive piglet, the producer of finishers risks losses due to unexpected declines in the price of a finisher. Moreover, buying expensive piglets and selling finishers at low prices results in a lack of liquidity for agricultural producers [1–5]. Sometimes it happens that piglets are bought at low prices and they are later sold in high prices as fattening pigs, but it all depends on the timing of the piglet purchase and the market situation, which is volatile [6].

The problem of profit variability from price fluctuations is having an increasing impact on the operation of farms, increasing the number of problems they face. These include the COVID-19 pandemic [7–9], the African swine fever (ASF) epidemic [2,10,11], increases in input prices [12], and legal considerations [13,14]. All of these factors increase production costs. Of particular relevance in the context of the European swine industry are the Green Deal initiative and the related Farm to Fork strategy, which promote greener and more sustainable agriculture and food systems [15]. Arising from all these factors, the problem of the instability of financial results makes it difficult to operate farms, especially the semi-subsistent ones that dominate the Polish production structure [16,17].

The problems described above have serious effects on Poland, which is one of the largest pork producers in Europe. In 2021, Poland accounted for 7.9% of pig production in the EU and ranked fourth among EU countries by volume [18]. Thus, it has a significant impact on the supply of this product in the EU. Profit variability poses a significant threat to the sustainable development of the meat sector, both in Poland and in other countries that produce similar volumes of pork livestock. This problem has been recognized for some time, but there has been a failure to implement solutions. Because of this, large corporations that conduct contract fattening account for almost 50% of the entities involved in pork production [4,19,20]. Contract fattening has negative consequences for market participation by smaller farms, which are more capable of realizing the important idea of shortening the supply chain [4,19,20]. This is particularly important in the Polish context where small farms dominate. In Poland, small farms are able to produce about 30% of standard output, while in the EU it is only 12%. According to the Polish system for the collection and use of farm accounting data network (FADN), small farms are defined as those with an economic value less than EUR 25 000 or 8 ESU or with an economic size equal to zero. The continuing unstable situation on the pig market in Poland for many years, the relatively small, low-performance farms, and the frequent fluctuations of the industry abroad are the factors that limit and often rule out profitable pig fattening on the farmer’s own account. Polish farmers struggle to develop their farms because their small family herds are unable to accumulate capital and invest.

Studies of pig production have shown that such volatile conditions make the profitability of pig production highly variable [3,5,21,22]. Therefore, solutions are being sought to reduce the effect of price fluctuations (to ensure stability) and guarantee a positive outcome (i.e. positive profit) for producers of piglets and finishers in each period [23]. Research on livestock efficiency has focused on livestock farms. Scientists initially studied the efficiency of dairy farms [24,25]. Sharma [26], on the other hand, studied the efficiency of pig production, and his results indicate significant production inefficiency among the pig producers studied. Other researchers have also reported similar results [21,27,28]. The conclusions presented in the analyzed studies allow us to conclude that the efficiency of pig production is determined by objective conditions (e.g. the level of costs in the market), which are rarely negotiable. Therefore, efficiency can be increased through the use of innovative solutions, including management and organization of contacts with suppliers and customers.

The purpose of this article, therefore, is to propose and evaluate a new solution that ensures the medium-term profitability and stability of the operations of pork livestock producers. In this regard, an innovative tool for the distribution of surpluses between producers of finishers and piglets is presented. This tool, hereinafter called the Algorithm, is based on shifting the settlement of sales of piglets to the date when the finishers are sold. This helps to avoid situations in which the entire chain surplus is taken over by only one of the parties, while the other makes a loss. The tool is based on cooperation between producers of piglets and finishers on terms that allow them to increase the stability and long-term profitability of their operations. The Algorithm appears to be a more beneficial alternative than the contract fattening model, while it provides a basis for sustainable integration in the pig production sector.

Previous studies have focused on profitability in the context of feed price fluctuations or on profitability factors, but without considering the important problem of price fluctuations [3,5,21,23]. We focus on the divergence of fluctuations in porker and piglet prices and the instability of profit that results from this divergence. The study of profitability has been reduced to an analysis of cost-effectiveness, as it is assumed (sometimes tacitly) that revenues cannot be changed, so ensuring profitability is only possible by reducing costs. We advocate that at least the variability of revenues can be decreased. In the analyses conducted so far, fewer factors were taken into account than in the study presented here—our solution is therefore more comprehensive. This study is part of the thread of considerations concerning the operation of the pork livestock sector, in particular the generation and stability of profit and, consequently, profitability over the medium term. This is an issue that concerns many economies, and EU countries in particular [29,30].

However, in terms of costs, our study is of a novel nature because, until now, studies have usually focused on the cost of feed [3,5,21,23]. However, the Algorithm considers other cost categories as well (e.g., utilities, veterinary costs, transportation), making it more like reality. For presentation purposes, we assumed these other costs as constant. However, the idea is that other cost should be updated on a regular basis based on the agreement between parties of the contract. Significantly, the solutions proposed in the literature were mainly microeconomic in nature (analysis of specific farms and not integration links) [17,21]. In contrast, the Algorithm takes into account the integration and cooperation of both the piglet producer and the finisher producer, making it systemic in nature. To ensure that integration between producers will last in longer period we assume that both parties should sign an official agreement that they will use Algorithm in their settlements.

We conducted our study for the Polish pork sector for the period January 2017–July 2022 on a weekly basis. The statistical analyses used data for 2017–2022, as one of the assumptions of the research carried out was to assess the situation of pig producers during the crisis. According to a report by the Institute of Agricultural and Food Economics of the National Research Institute (2023), the Polish pig market is experiencing a number of changes in the structure of domestic breeding. In addition, since 2015, the market has been facing problems such as ASF, increased production costs, changes in animal husbandry regulations and competition with imported products [31]. The aforementioned factors prompted the researchers to analyse this time frame in order to be able to determine how the crisis in the pork market will affect the profitability of pig production. The upper limit of the range was set by the need to present the findings (the results were analyzed in August 2022). The analysis is based on the prices of agricultural products, including the prices of finishers, piglets, and crops, as well as information on other costs related to the production of finishers and piglets. The sources of the data are official quotations from the Polish Ministry of Agriculture, the German pig exchange (VEZG), the Danish piglet exchange, the Agrolok company for crops, and the official exchange rate of the Polish Central Bank (NBP). The data were used to assess the market situation, diagnose the relationship between several variables (finisher prices, piglet prices, production costs), design a profit stabilization tool for the finisher producer, and evaluate the performance of this solution. We analyze this performance using basic statistical measures such as coefficient of variation, standard deviation, and mean. They were applied to verify the link between the prices of piglets and finishers, thus validating the logic of the Algorithm by changing the formula for the settlement between the producers of piglets and finishers. As part of the evaluation of the solution, the pattern of profits under current market conditions was compared with hypothetical conditions if both types of producers used the Algorithm to settle.

The rest of this article is organized as follows. In the next section, we present the theoretical basis for the proposed mechanism, referring primarily to transaction cost theory. Then, we discuss the evolution of the pork sector in the EU and the most popular ways to organize production. We then focus on the detailed assumptions of the Algorithm and provide an overview of data sources. Next, we present the hypothetical effects of the Algorithm in 2017–2022 based on the example of the pork sector in Poland, comparing those results with the actual market situation. Finally, we provide the main conclusions and recommendations for agricultural policy. The analysis showed that implementing the Algorithm would reduce the variability of a finisher producer’s profit by 45% in 2017–2022 (the standard deviation drops from 19,28 to 10,62 EUR/unit while keeping the average profit constant. The number of periods with negative profit decreases significantly compared to the current situation.

## Theoretical framework

According to transaction cost economics (TCE), economic activity is associated with coordination costs, which result from the fact that many economic agents operate in the market simultaneously [32]. These include the costs of collecting market information, the costs of management and contracting (including the costs of negotiation), and the costs of controlling the day-to-day execution of contacts. According to this theory, economic actors, such as farms, should choose organizational forms and institutions that minimize exchange costs [33]. Transaction costs are a function of three elements: the frequency of transactions, uncertainty, and the specificity of products and assets [34]. The greater the level of uncertainty and asset specificity, and the lower the frequency of transactions, the greater the transaction costs [35].

Agriculture, including pork production, is a sector where transaction costs are relatively high, because the number of transactions between farmers and purchasers is relatively small, while the assets held by farmers are highly specific and cannot be easily reallocated to other uses. For example, machinery used in a fattening plant can only be used for this purpose. A distinctive characteristic of the agricultural sector is the high level of uncertainty, which includes uncertainty about price conditions (the problem of strong price variability) and production conditions. In pork production, this is manifested, for example, in the variable number of piglets per farrowing or the variable number of finishers ready for sale. There is also institutional and policy uncertainty, which results, for example, from the pressure to raise quality standards for meat production and the pressure to reduce meat consumption.

As Ménard [36] points out, in conditions of increasing uncertainty and high asset specificity, transaction costs increase faster for common market contracts. In the case of hierarchical governance structures, such as various forms of horizontal and vertical integration, the increase in transaction costs is slower. These different governance structures in the agri-food sector are de facto institutions in the sense of the new institutional economics (NIE), as they determine the bargaining power of the parties involved in a transaction [37,38].

The desire to reduce transaction costs may become the main incentive to merge into producer groups and to explore opportunities to shorten the supply chain through vertical integration. Indeed, pig producers, most often of a small-scale nature, being the initial link in the marketing chain, have a limited ability to influence sales prices. This is related not only to the proximity to the final buyer but primarily to the low economic power of individual entities. This contributes to setting transaction terms more favorable to stronger partners, such as processing plants. Indeed, in the case of the latter, we generally deal with an oligopoly.

To improve their negotiating position, farmers sometimes decide to produce livestock collectively to improve their bargaining (negotiating) power and obtain better prices and contract terms. This is particularly important during a downturn in the pork sector, as it prevents such sharp and abrupt drops in purchase prices, as in the case of individual farms. Simultaneously, cooperation within a producer group generally leads to more efficient breeding (by reducing the costs of machinery and equipment and the costs of supplying inputs and distribution, increasing labor productivity, and improving technology as a result of sharing knowledge and experience). In this way, the negative consequences of the “up” in piglet prices are mitigated by reducing costs [39,40]. Besides, thanks to cooperation, the risk of fluctuations in supply and prices is spread among many group members, and competition between them is reduced. This serves to achieve higher prices at sale. Empirical research conducted by Ciliberti et al. [41] on Italian farms confirmed that specialization of assets and variability in the level of production were important determinants of farmers’ involvement in horizontal cooperation.

The presence of transaction costs is also a rationale for contracting between entities within the framework of vertical integration, resulting in reduced risk and a change in the degree of ownership specificity. There are many empirical forms of vertical integration in the pork sector, such as spot market transactions, which can be supplemented by formal and informal long-term agreements that specify the terms of sale and purchase of raw material (contract fattening), and the full vertical integration that includes retail trade. Cooperatives are an advanced form of vertical integration in which farmers participate not only in the production but also in the distribution and sale of products, thus realizing greater added value. Many European countries (e.g., Germany, France, Denmark, Austria, Spain, and Italy) have systems for setting pig prices by intentionally established organizations, such as the German VEZG exchange. These prices are respected by both parties of the contract—farmers and processing plants—and herds do not need to use contracts that are subject to many rigors, including the fixing of delivery prices. In Poland, contract fattening is becoming increasingly popular because production risks are shifted to the fattening organizer [42].

Why do many agricultural producers, especially in Central and Eastern Europe, not participate in any form of horizontal or vertical integration? TCE points to agents’ opportunism and limited rationality [43]. The latter, however, is assumed to be due mainly to cognitive limitations. Traditional TCE, however, has been criticized because it ignores problems of social embeddedness, including trust [44]. These elements may be particularly relevant in the agricultural sectors of countries that are experiencing various forms of forced collectivization. According to Czyżewski et al. [45], the problem lies not only in the cognitive limitations of farmers, but also in the perceptual limitations described by the theory of planned behavior through elements such as attitude toward the behavior, perceived social pressure, and perceived ability to perform the behavior (perceived behavioral control) [46]. While cognitive limitations can be reduced by proper education of farmers (improving human capital), reducing perceptual limitations requires increasing social capital and trust with each other. Higher levels of social capital not only increase the likelihood of cooperation, but they can also help realize the farm’s main goal of improving its economic performance [47].

Thus, an effective tool for stabilizing the economic situation of producers in the pork sector should guarantee the regularity of producers’ income, promote the horizontal integration of pork producers, and strengthen various forms of vertical integration. Using the Algorithm will also be facilitated by a higher level of education of its potential users. Given the limited level of trust among farmers, especially in post-socialist countries [48–51] it is important not only to present farmers with the financial benefits of implementing the proposed solution but also to build a network of mutual trust that fosters cooperation.

## Profitability of pig production

The Polish pig sector is still characterised by a high fragmentation of domestic production, which differentiates the raw material and makes it less suitable for the production of even batches of pork products, limits the standardisation of production, raises the costs of production and purchase of livestock. Despite the fact that in recent years it has been possible to observe processes of concentration of pig production, the average size of the pig herd in Poland is still significantly lower than in countries that are European leaders in pork production. One of the factors hampering the development of Polish production is the sector’s dependence on imports of animals for fattening. There is increasing competition on the European market, which has led processing companies to import piglets to Poland, mainly from Denmark and the Netherlands. In addition, most processing companies import large consignments of half carcasses to a uniform standard, from other EU countries, to meet market expectations. Every year the Polish market imports approx. 700 000 tonnes of pork. This negative trend, making our pig sector dependent on piglet imports (mainly from Denmark), has been growing since 2008. Piglet imports now account for almost 30% of the annual slaughter. Producing more than 6.2 million piglets in a year requires 200,000 sows. An important issue is the size of the farms where additional sows would produce piglets in the country. This is related to both the economics of production and the possibility of populating fattening facilities from a single source (epizootic considerations) [4].

The key challenges of the pig sector remain the profitability of production and variability. The unstable profits of pig producers result mainly from constant fluctuations in supply, the prices of livestock, and the raw materials needed for feed production.

Pork production in 2022 in the EU was 22,4 million tons, and the pig sector accounted for 8.5% cent of EU-27 agricultural production, more than any other meat sector [19]. Pig production is dominated by Spain, Germany, France, Poland, the Netherlands, and Denmark, which supply more than half of the pork produced in the EU (Table 1). Although pig meat production was relatively stable between 2018 and 2022, there was an 20% decrease in Poland, which was related to fluctuations in the profit prices of the producers of piglets and finishers, as well as the development of ASF.

**Table 1:** Pigmeat production in the EU-27, in 1 000 tonnes live weight.

Countries	2018	2019	2020	2021	2022
Spain	4 530	4 641	5 003	5 168	5 254
Germany	5 350	5 232	5 118	4 964	5 075
France	2 182	2 200	2 201	2 190	2 140
Poland	1 973	1 866	1 853	1 869	1 645
Netherlands	1 536	1 628	1 662	1 770	1 570
Denmark	1 583	1 500	1 597	1 665	1 685
Italy	1 487	1 464	1 286	1 325	1 322
Belgium	1 073	1 039	1 099	1 252	1 144
Hungary	467	462	456	476	474
Czechia	220	219	221	229	218
**EU-27**	**23 156**	**22 996**	**23 261**	**23 662**	**22 425**

Source: [16,18].

Over the last few years, trade in pig livestock between EU countries has been affected by sanitary conditions, logistical difficulties related to the COVID-19 pandemic, and the fact that production is subject to stricter regulations and increased competitiveness. In 2021, pig trade between EU countries was 10% lower than in 2017. The EU pig sector, in terms of trade, was mainly affected by the spread of ASF in Belgium in 2018 and then in Germany in 2020 [16,52–54].

The EU pig sector is currently in a situation of oversupply due to increased production and reduced purchases by China [12]. This oversupply keeps applying downward pressure on prices. The year 2021 continued the downward trend in pig prices that began in May 2020. In the first quarter of 2021, the average price of Class E pigs in the EU-27 was EUR 136.81/100 kg slaughter weight, 27.3% lower than a year earlier. In Poland, the price of a pig with the same parameters was EUR 129.67/100 kg, which was 30.8% lower than in Q1 2020. The decline in pig prices was the result of a slight increase in production in the EU27 and a reduction in demand linked to the COVID-19 pandemic in the HoReCa channel [55]. The pandemic accelerated the fall in prices, which would probably have happened anyway (the pig cycle), but a few months later. In this situation, the annual fall in pig prices occurred in May.

The key to increasing the long-term profitability of production is to raise the cost-effectiveness of outcomes for each step of the process of using available resources [56]. The prices of piglets and finishers play a major role in shaping the profitability of pig production, which is further determined by the cost of feed and other inputs used in agricultural production. Strong fluctuations in input prices, including feed prices, yield significant changes in the level of costs incurred by farmers, and this significantly worsens the profitability of pig fattening [21].

Market fluctuations affecting the pork sector have been given their own name: the “pig cycle” [27,28,57–59]. Its essence is that the profitability of animal production and the associated changes in real volumes are caused by the situation in the crop market. Harvest volumes shape crop prices and influence the relationship between the purchase price of livestock and feed prices. This relationship is taken into account by livestock producers when deciding on production volumes. Crop harvests are seasonal and happens once a year, and the herd supply is characterized by periodic fluctuations. With a relatively constant demand for pig meat, this leads to fluctuations in the purchase price of livestock and changes in the profitability of production. Pig farmers, who are also input price takers, decide to use scarce feed, labor, and land resources to increase livestock production and achieve the goal of maximizing profits. With the limits of resources and environmental constraints, the development of the pork industry cannot be guaranteed by increasing inputs only. Production costs must be minimized by rational allocation of factor resources in the existing input–output situation [21].

**Fig 1:**
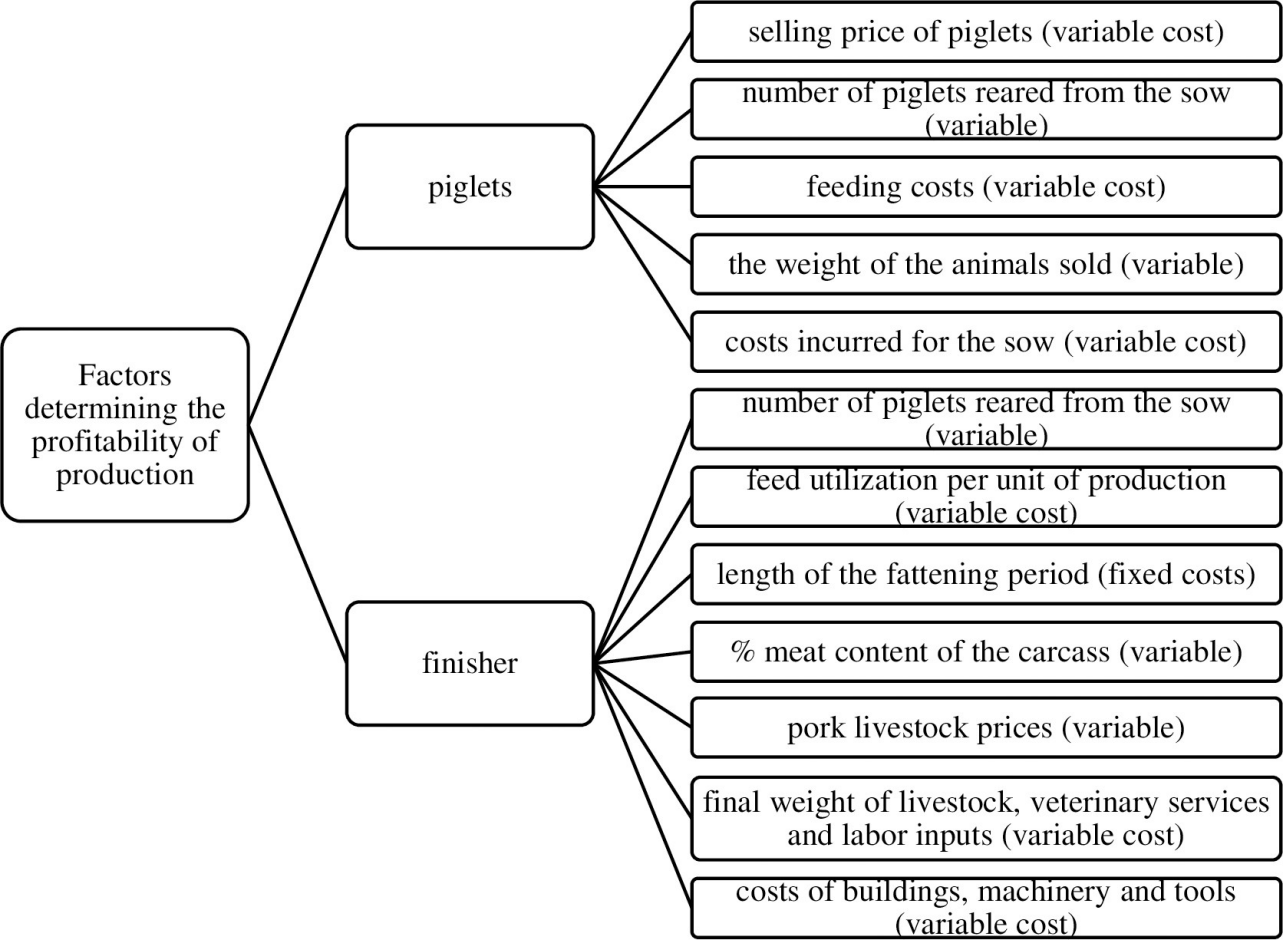
Profitability factors for piglet and pig production. Source: Own elaboration.

Changes in the pig sector force producers to continuously analyze all the factors shaping the profitability of production so that they can react to unfavorable changes in real time and offset them (Fig 1) [5]. Fig 1 below is the result of a literature search and does not refer to the variables used in the Algorithm calculations. This figure relates to the issue of the subject of production profitability in general.

The greatest influence on the profitability of piglet production is the number of piglets raised from the sow. So, improving farrowing frequency increases economic fertility. This indicator can be improved by changing the organization of production, balanced feeding according to the age and needs of the animals, ensuring correct microclimatic parameters in the shed, and maintaining the health status of the herd. From an economic point of view, the service life of the sow also is of great importance. A well-prepared sow, kept in appropriate microclimatic conditions, should lay at least 5 or 6 numerous litters [60]. As the number of animals kept increases, the cost of producing a piglet or a kilogram of livestock decreases. On the other hand, the price per unit of production (the possibility of selling a large number of balanced batches of the product) and profit from sales increase [21,61].

The profitability of pig production depends on several factors. Some of them involve the producer (e.g., feed use, economic prolificacy), and some are not influenced much by the farmer (selling price, feed price, and market prosperity). Costs related to animal nutrition and feed use very often influence the profitability of production. One important element is the scale of production carried out (i.e., the volume of sales). The larger the production, the lower the unit cost of production. The health status of the herd, the zoohygienic conditions, and the obligations arising from adapting the farm to changing environmental requirements will soon play a huge role in shaping the profitability of pig livestock production in the EU [55].

From an institutional point of view, the profitability of pig production is also influenced by the organization of the pig market [62–65]. There are four main ways to organize pork production. The first is the production of pigs by independent producers, who remain free to choose their suppliers of piglets and production materials, and their buyers of finishers. The second, more formalized way to organize pork production consists of contracts between farmers and buyers, providing delivery of pig livestock at a fixed time and price. The third approach involves vertical integration contracts. In this model, the farmer brings his land, buildings, and labor into the integrated system. The company (integrator) transfers sows or piglets, feed, and medicines to the farmer for temporary management; ensures the purchase of the piglets or fattening pigs produced; organizes transport, marketing, and sales; and secures cost-free veterinary support, services, and medicines. The integrator also provides ongoing training to the producers. The farmer, in turn, receives contractual remuneration for the animals produced. The fourth option is the collective sale of pigs through cooperatives. Pig production and sales by cooperatives is the most significant form of production organization in France and Denmark. Cooperatives are also often involved in slaughtering and processing.

In Poland, producers mainly follow the first approach to production, and they use two production cycles of pig farming: closed and open [60,66]. In the closed cycle, a farm maintains a core herd of sows to produce piglets for fattening. In the open cycle, farms are divided into two types. In the first stage, the farmer maintains a core herd of sows and produces piglets for sale. In the second, the producer buys piglets that are then fattened to a certain weight [19,20].

The specificity of each kind of production cycle determines the level of costs incurred by producers. The specificity of each kind of production cycle determines the level of costs incurred by producers. In a study by Zhao [12] conducted in the Chinese pig market, it was shown that the cost of producing a piglet in a closed cycle is generally lower than the cost of purchasing it for open-cycle fattening. Farmers producing in a closed cycle are therefore not dependent on piglet market prices, making this type of activity more profitable when piglet prices are high. The highest costs incurred by producers are feed costs, which can account for up to 75% of pig maintenance costs. Feed costs are mainly determined by cereal prices. The higher the prices of cereals and other feed components, the higher the cost of feeding the animals, and the lower the potential profit for the farmer [12]. This relationship is also confirmed by agricultural calculations carried out in the time range 2015–2019 by the Agricultural Advisory Center in Poznań, where it was also found that the more profitable cycle was the closed cycle compared to the open cycle. Therefore, it can be presumed that a closed cycle is more favourable for the profitability of pork livestock production. It is worth implementing solutions to integrate piglet and fattening pig producers. The first step in this process could be to conduct transactions between these market participants in such a way that the financial outcome is favourable to each party regardless of the economic situation [67].

Problems related to fluctuations in profit and profitability are causing an increasing number of Polish farms to withdraw from the traditional production model without any formal integration and opt for contract fattening, but this is not an optimal model. By participating in contract fattening, pig fattening farms terminate cooperation with their existing (usually domestic) piglet suppliers. Contract fattening also causes unfavorable changes in the structure of producers. These changes include strengthening the dominance of large corporations and the disappearance of the fattening tradition of smaller, domestic farms that can offer a higher-quality product [19,20]. Furthermore, if the cooperation between the farmer and the purchaser is reduced to a simple contract for the delivery of fattening pigs (without real vertical integration with the farmer’s share of the integrator’s profit), this form of production protects the farmer from financial loss, but it closes off the possibility of achieving a higher profit. Studies have shown that contract fattening provided lower income (family labour remuneration) when compared to other forms of pig market organisation in Poland [68]. Therefore, we assume that although the aim of contract fattening is similar (stabilization of the market), its consequences may not be desired from the general market perspective as well as from the perspective of a single producer.

Despite the growing popularity of contract fattening, many Polish farmers still prefer flexibility in selecting suppliers and buyers. Even if producers do not wish to set up producer groups or cooperatives immediately, it is still possible for individual producers of piglets and fattening pigs to cooperate, e.g., where they have carried out regular market transactions between themselves in the past. Such micro-trading relationships are an important determinant for the feasibility of implementing the Algorithm. Taking into account that Polish producers are used to working with specific counterparties, we believe that the Algorithm can easily become a popular tool on local pig market. We are aware that implementation of such a tool in more integrated settings in western Europe can be limited, but the proposed tool is designed mainly for agricultural context in Central and Eastern Europe.

The issue of pig production profitability in the literature is often analyzed together with the problem of efficiency (cf. S1 Table). This is because of the assumption that market conditions (including prices) are hardly negotiable. In this case, the profitability of pig production can be raised mainly by increasing efficiency understood as the ratio of output to the inputs incurred. That is why we refer to studies that address not only profitability itself but also profitability in relation with efficiency.

Sharma et al. [69] found significant production inefficiencies among the pig producers surveyed. Other researchers [3,5,21,23,70,71] have indicated that the pig sector is characterized by low short- and long-term profitability of production, and these are associated with fluctuations in profit for piglet and finisher producers. To confirm their theses, the researchers used two methods: stochastic frontier analysis (SFA) and data envelopment analysis (DEA), using the variables of finisher and piglet production volume, finisher and piglet production costs, and feeding costs.

The conclusions of those researchers indicate that the profitability of pig production is determined by objective conditions (e.g., the level of costs in the market). Therefore, efficiency is perceived as the main way to improve profitability. This can be achieved by using innovative solutions, including the management and organization of contacts with suppliers and customers (i.e., different forms of horizontal and vertical integration). In this context, the Algorithm proposed in this paper is an extension of previous attempts to solve the issue of lack of profitability. Our solution fills the research gap in analyzing and finding ways to overcome the profit fluctuations of pig sector participants, as it proposes an innovative method of sharing the surplus between the piglet producer and the finisher producer.

## Data and methods

### Data

The sources of the data used in the study are specified in Table A1 in S1 File. The finisher prices are taken from the Ministry of Agriculture databases and listed on a weekly basis.

The piglet prices are taken from the Danish Stock Exchange (SPF). The calculations include the variant of piglets with the highest health status without porcine reproductive and respiratory syndrome virus (PRRS). We decided to use these prices due to the large impact of import of Danish piglets on Polish market. The standard practice is that piglet traders add some margin on Danish price. After consultation with farmers we decided to set this margin on the level of EUR 13.66/unit. Using the exchange rates of National Bank of Poland, we recalculate data for piglet price in Danish krone (DKK) and margin in EUR to polish zloty (PLN). Therefore, all prices used in the Algorithm are finally in PLN. However, to make the detailed presentations of the Algorithm for international reader more convenient, we present an example (S1 Fig) using EUR. It can be raised that the problem of exchange rate (in particular DKK to PLN) can influence the operation of algorithm. However, in the studied period it fluctuated between 0.56 and 0.65 PLN per DKK. Standard deviation was 0.02 and coefficient of variation was 3.7% only. The PLN/EUR exchange rate (used for margin calculation) was also quite stable. It fluctuated between 4.15 to 4.84 with standard deviation equal to 0.16 and coefficient of variation equal to 3.6%. Therefore, we can conclude that the exchange rates were rather stable, and they shouldn’t have a big impact on the results.

The prices of cereals used for feeding are taken from the average monthly data of the Ministry of Agriculture and the daily stock exchange quotations of Agrolok. The remaining operational costs (veterinary costs, utilities, labor, and transport) were assumed at a constant average level established on the basis of the reference methodological publication of the Danish research and development organization called “Seges Innovation” [72] and a manual on pig farming [73]. The findings from these two sources were compared to the experiences of Polish pig producers to verify that the adopted standards correspond to Polish conditions. The same sources were used to determine the optimal feeding model, which is important for calculating feed costs. Assumptions for calculating labor costs in piglet and finisher production, as well as piglet transportation costs, are presented in Tables A2, A3, and A4 in S1 File, respectively. The costs of falls are variable—the assumptions for their calculation are shown in Table A5 in S1 File.

Table A1 in S1 File gives the original units of the variables. In the following paper, all prices, costs, and profits have been converted from PLN to EUR at the respective exchange rate for results presentation purposes.

### Methods

The research objective is to evaluate an Algorithm that reduces the negative impact of price fluctuations on the long-term profitability and stability of pork livestock producer enterprises. To achieve this, the following steps were taken:

1. The current situation was presented, focusing on the amount and variability of the profit of the producers of finishers and piglets and the evolution of prices—the trends of changes in these categories were compared.
2. The principles of the functioning of the mechanism, which is supposed to stabilize profits in pig livestock production, are presented in detail, supplementing the description with numerical examples.
3. The Algorithm was evaluated using statistical methods. The development of profits was compared for the current situation and that proposed by the Algorithm. The variables were analyzed on the basis of trends of change, averages, and coefficients of variation. The stability of farm operations was compared both with and without using the Algorithm.

The study was conducted in the Polish pork livestock sector. Weekly data from 2017 to 2022 were considered.

### Algorithm as an innovative method—assumptions and their rationale

Due to price fluctuations and the strong correlation between piglet and finisher prices (“ups” and “downs” in the pig industry), it is quite common for a finisher producer to incur a loss (buying expensive piglets and selling finishers at a low price), resulting in a lack of liquidity for entrepreneurs. Against this background, we propose an innovative method, the Algorithm, which reduces the effect of price fluctuations (ensuring stability) and guarantees a positive financial outcome (or minimizing loss) for both types of producers in each period. The Algorithm leads to a stabilization of piglet prices based on the price of a piglet at the time the finishers are sold to the slaughterhouse. The settlement of the purchase of piglets takes place in the week of the sale of the finishers instead of 14 weeks earlier, as is currently the case. The Algorithm can be implemented on the basis of an agreement (vertical integration) between piglet and finisher producers, so it is based on close cooperation between the two types of producers. This is similar to the Danish model for organizing pig production [65] (Thomsen, 2022). Denmark, which is an important producer of pork in the world, has a long tradition in this production. In Denmark, as in Poland, there is close cooperation between farmers and slaughterhouses, which has contributed to the development of trade relations between piglet producers and fattening farms. In Denmark, there is a cooperative system in the pork market, which includes pig breeding, the feed industry, slaughter and distribution. All activities of the cooperative are coordinated by an organization called Danske Slagterier, which collects information used in: pig breeding to select the best breeds, live pig production, organization of an appropriate breeding system, and slaughterhouses for the appropriate sorting of pork [65]. Following the example of activities undertaken in Denmark, the Polish company Agrointegracja tries to bring together farmers producing pigs and help them develop their farms and increase profits from their activities. The described algorithm is similar to the Danish model, as it takes into account the price variability of piglets and finisher.

To mitigate the profit fluctuations of the finisher producer, we based the Algorithm on two assumptions:

1. A change to the principle of settlement between the finisher and piglet producer: Instead of a 14-week interval, it takes place on the day the finisher is sold. This eliminates the impact of price fluctuations on the profit of piglet and finisher producers in the pig cycle.
2. Different cost categories are taken into account when calculating the profit of the piglet producer and the finisher producer: feeding costs and other operating costs (veterinary costs, farming overhead, transportation costs, falls, and labor costs).

Table 2 compares the Algorithm with the current situation. First, in the current situation, the settlement between the finisher producer and the piglet producer takes place on the day the piglets are purchased (14 weeks before the finisher is sold). Under the Algorithm, the settlement with the piglet producer takes place on the day the finisher is sold. Second, the profit of the two types of producers is calculated separately, whereas the Algorithm calculates a joint profit (the finisher producer’s revenue minus all costs related to growing the piglets and finishers). This is then divided between the two types of producers according to a *predetermined proportion*. Third, under both the current situation and the Algorithm, the basic production assumptions remain the same, including that fattening lasts 14 weeks, costs and income are based on market conditions, and the average profit of both types of producers remains the same.

**Table 2:** Comparison of the current situation with the algorithm.

Current situation	Algorithm solution
Costs and revenues are calculated based on market prices.	
Finisher and piglet producers generate profit separately.	A total profit is calculated and then divided according to calculated proportions.
Full settlement between finisher and piglet producers takes place on the day of piglet sale.	Piglet producer receives a lump sum of PLN 180/unit on the day of piglet transfer to cover operational costs; full settlement takes place after 14 weeks.
Average profit remains at the same level.	

Source: Own elaboration.

In the Algorithm we determine the optimum ratio for sharing the surplus generated over the entire production cycle between the piglet and finisher producer. Profit-sharing ratios are always determined on the basis of weekly historical data from the beginning of 2017 (comparable price and cost data have been available since then) until the week of settlement. The period is therefore continuously extended, so that the reliability of the calculated values is increased. For this article, the period January 2017–July 2022 was considered. The upper limitation of the range is due to the need to analyze the results, which were compiled in August 2022.

**Fig 2:**
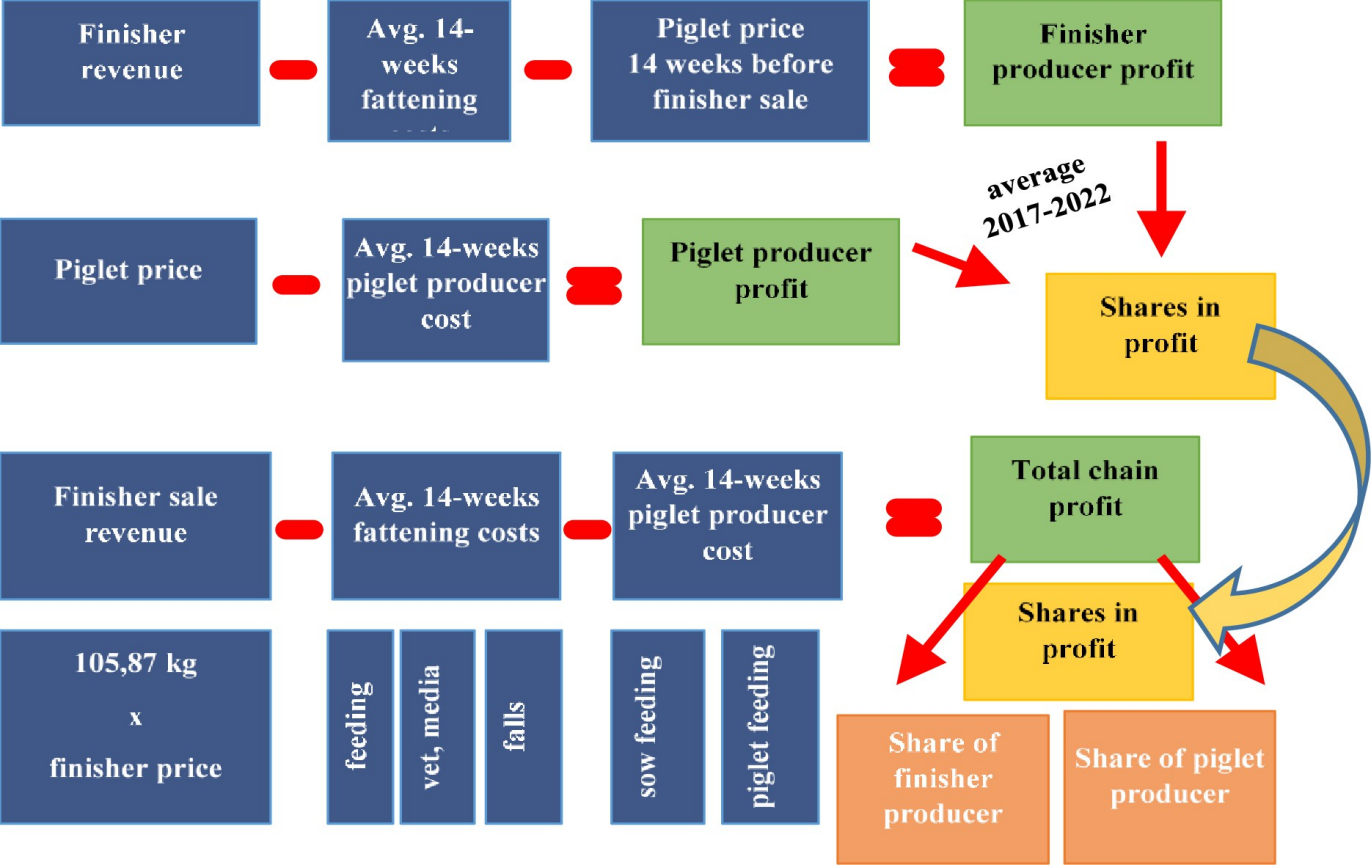
Calculation of profit-sharing proportion and settlement formula. Source: Own elaboration.

The Algorithm uses the following procedure (Fig 2):

1. The profits of the producers of finisher and piglet according to the market situation and the assumed production standards are calculated, taking into account the revenue at the time of sale and the average costs for the 14 weeks preceding the sale. (Details of costing and production standards are below.) The profit per unit of finishers and piglets is analyzed.
2. Average values of the above categories are determined for the period from the beginning of 2017 to the week of settlement, in this case January 2017–July 2022.
3. The profit for the whole chain is calculated as the difference between the revenue from the sale of the finisher and the production costs of the piglets and the production costs of the finisher.
4. The (average) share of each producer type in the profit for the period from the beginning of 2017 to the week of settlement, in this case January 2017–July 2022, is calculated as the quotient of the average profit for each producer type to the profit for the whole chain. The data are continuously updated and added to the weeks (the start of the time range is always January 2017), so the ratio changes depending on the week of settlement.
5. Profit across the chain is shared between producers according to the above-determined proportions. For example, for the period January 2017–July 2022, the proportions of profit sharing are as follows: 44% of the profit in the whole chain for the finisher producer and 56% for the piglet producer. Importantly, if a producer type achieves higher cost efficiency than the assumed production standard, the additional surplus (over and above that determined on the basis of the standard) is not shared. It remains with the producer, who achieved higher than assumed efficiency. In this way, the Algorithm should not diminish the interest of both types of producers to improve production efficiency. It is also worth noting that the piglet producer usually takes a larger share of the surplus, which is his reward for waiting 14 weeks for settlement (instead of receiving the surplus immediately, as is currently the case).
6. The settlement with the piglet producer is as follows: On the day the piglet is sold, the piglet producer receives a lump sum to cover his costs according to the average historic cost of production, and the profit settlement takes place on the day of the finisher sale. This ensures that the finisher price and piglet price are derived from the same day, eliminating the impact of price fluctuations on the surplus.

The settlement shift is based, in part, on the Spanish model of pig livestock production [63]. Also, here, certain production standards are adopted, and the ability to generate a surplus higher than that implied by the assumptions creates a kind of “efficiency bonus.” The advantage of the Algorithm, however, is that it is based on market data, it allows producers to remain independent (instead of being linked on the basis of supplying inputs and receiving fattening pigs as in the Spanish solution), and the proportion of profit sharing is calculated on the basis of historical data over a relatively long period.

The Algorithm accounts for the variability of prices and production costs (including feeding costs) by analyzing historical data from the beginning of 2017. As already mentioned, we update the dataset on a weekly basis (*the start of the time range is always January 2017*), so the proportions change depending on the week of settlement. However, to present these results, the time range ends in July 2022. The standards for finisher and piglet production have been assumed to be at a level achievable for most finisher or piglet farms.

The assumptions and standards for finisher production are shown in Table 3. The return of the finisher pig producer is calculated as the product of the price of the pig at the time of sale and the HCW (hot carcass weight) weight.

**Table 3:** Assumptions in finisher production.

Category	Assumption
Piglet weight (kg)	30
Target live weight (kg)	134.01
Efficiency	0.79
HCW weight of a finisher of E class	105.87
Fattening time	14 weeks + 1 week of break between fattening periods
Feed conversion coefficient	2.74 kg of feed per kg of live weight gain

Source: Own elaboration.

The following are included in the finisher production costs: piglet cost, veterinary costs, utility costs (water and electricity), labor costs, falls, and feed costs. It is worth pointing out that in designing the Algorithm, an optimal feeding model was defined, which is based on the reference methodological publication of the Danish research and development organization SEGES [72] and a manual on pig farming [73], as well as the experience of farmers. The application of this feeding model guarantees the best possible use of feed raw materials and efficient fattening. Sources of other data are specified in the Data section. The detailed information on how the feed costs were calculated are provided in Table A6 in S1 File.

In terms of piglet production, 28 piglets are assumed at 2.14 cycles per year. The piglet producer’s revenue is determined as the product of the price at the time of the piglets’ sale and the weight of 30 kg.

The costs of piglet production include veterinary costs for the sow and piglet, utilities (water and electricity), transportation costs, labor costs, and feed costs. As in the case of finisher production, an optimal feeding model was identified, which is based on the reference methodological publication of the Danish institution SEGES [72] and a manual on pig farming [73], as well as the experience of farmers. The use of this feeding model guarantees the best possible use of feed raw materials.

The income and cost values determined in this way were used in the calculations conducted according to the procedure described above. Importantly, the tool is objective and automatic, with no possibility of third-party interference. This reduces the concerns of the integration participants regarding the fairness of the surplus distribution. Feed recipes and other solutions related to the production process (e.g., a negotiated input price) were assumed to be achievable for the average participant in the pig production sector.

## Results and discussion

### Profit of a finisher producer in the current situation

**Fig 3:**
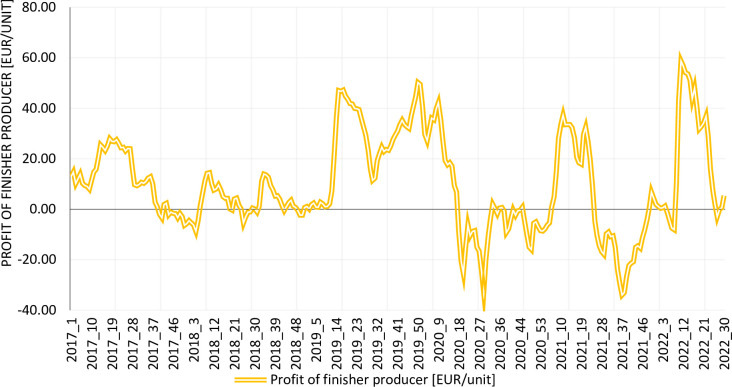
Finisher producer profit in 2017–2022 on a weekly basis. Source: Own elaboration.

The profit of a finisher producer is calculated as the difference between revenue (the product of the purchase price and weight, as indicated in the previous section) and production costs. Details are provided below. Between 2017 and 2022, the profit of the fattening pig producer was characterized by quite high variability, with the dynamics of change increasing, especially from the early part of 2019 (Fig 3). Long periods of negative or near-zero sales profit were observed, such as in the second half of 2020 and 2021. The high variability of profit makes the business highly uncertain, which is in line with observations made by other researchers (cf. the section on the Profitability of Pig Production).

**Fig 4:**
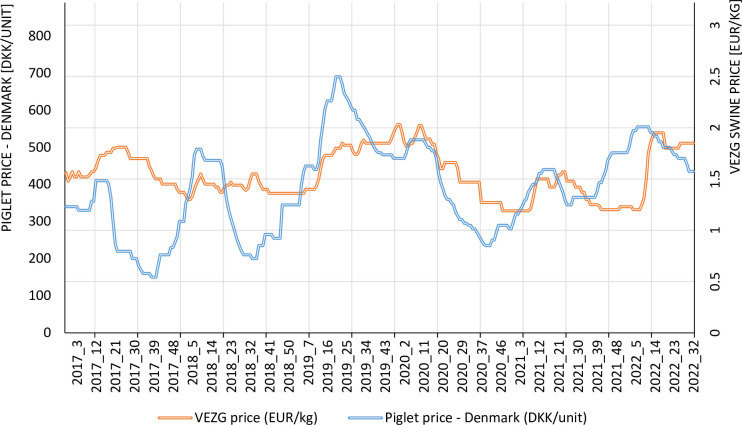
Piglet and finisher prices in 2017–2022 on a weekly basis. Source: Own elaboration.

According to the observations of the pig production sector, the main sources of large fluctuations in the financial results of a pig livestock producer are changes in the prices of finisher and piglets and the strong dependence of the piglet price on the finisher price. In particular, this is the period when the finisher price is high and the piglet price also is high. The problem, however, is that the finisher is sold around 14 weeks after buying the piglet. Fig 4 shows both the frequency and depth of the price fluctuations and the convergence of the trends in prices for piglets and finishers—especially at the beginning of 2019, all of 2020, the first half of 2021, and the first half of 2022. In connection with these two factors, situations in which piglets are bought at a high price and finishers are sold at a relatively low price are very likely and occur often.

The juxtaposition of the above observations makes it feasible to analyze the impact of price fluctuations on the long-term profitability of finisher production and the stability of conditions in the sector. In the current situation, the following conditions are assumed:

- The settlement of the finisher producer with the piglet producer takes place on the day of purchase of the piglets (14 weeks before the sale of the finisher);
- After 14 weeks, the finisher producer holds the sale;
- A 1-week gap is maintained between the 14-week periods of fattening.

As already mentioned, due to price fluctuations and the strong correlation between piglet and finisher prices (“ups” and “downs” of pig prices), it is quite common for a finisher producer to suffer a loss (buying expensive piglets and selling finishers at a low price), resulting in a lack of financial liquidity.

**Fig 5:**
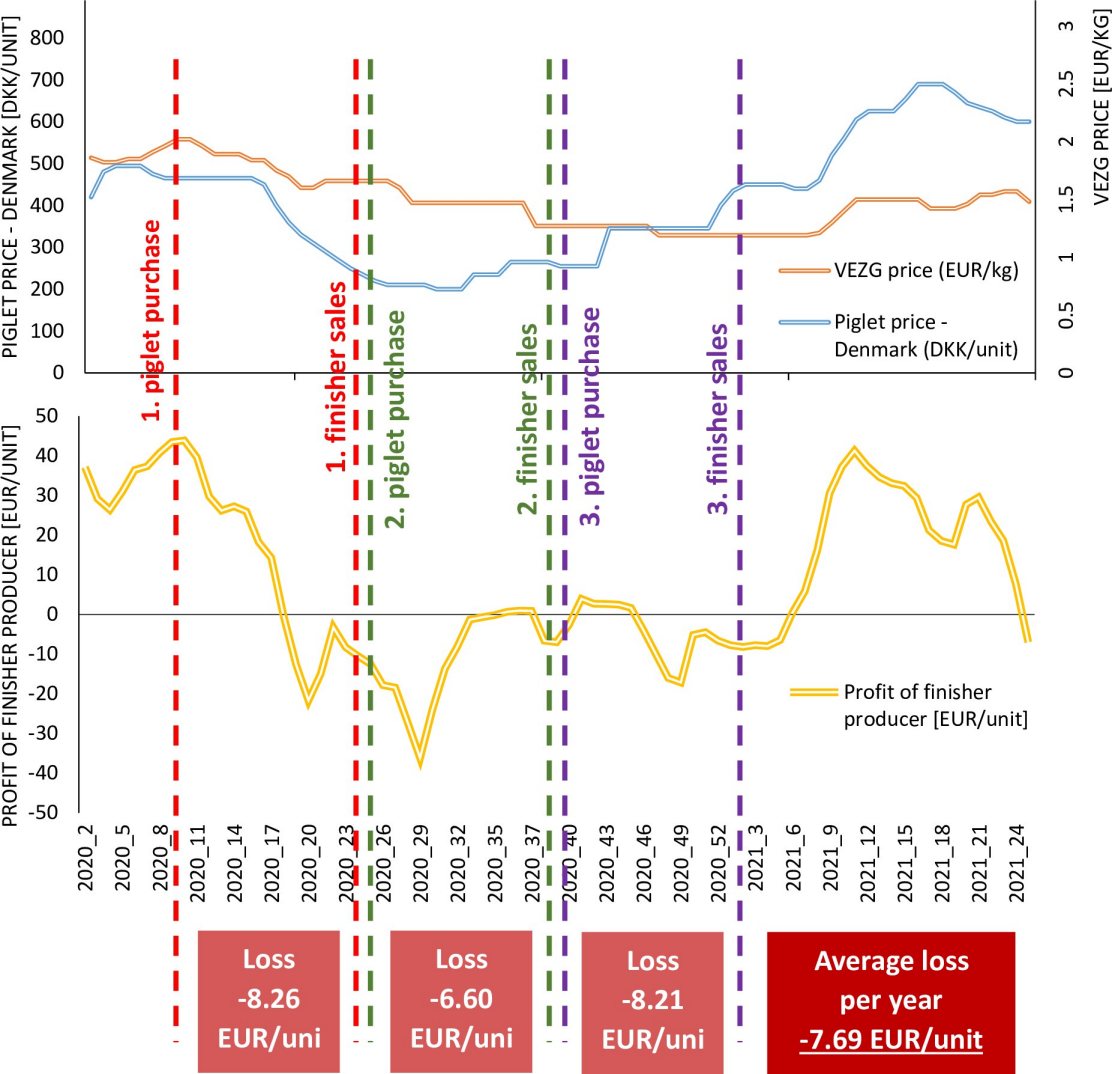
Profit from finisher sales in 2020. Source: Own elaboration.

Such a situation is illustrated in Fig 5, which shows the financial results from almost a year’s activity in finisher sales in 2020. This period was chosen for analysis because it was characterized by particularly strong fluctuations in profits from finisher sales. No positive financial result was achieved in any of the fattening periods analyzed, while the average loss from the activity in 2020 was more than EUR 7.5 per unit.

**Fig 6:**
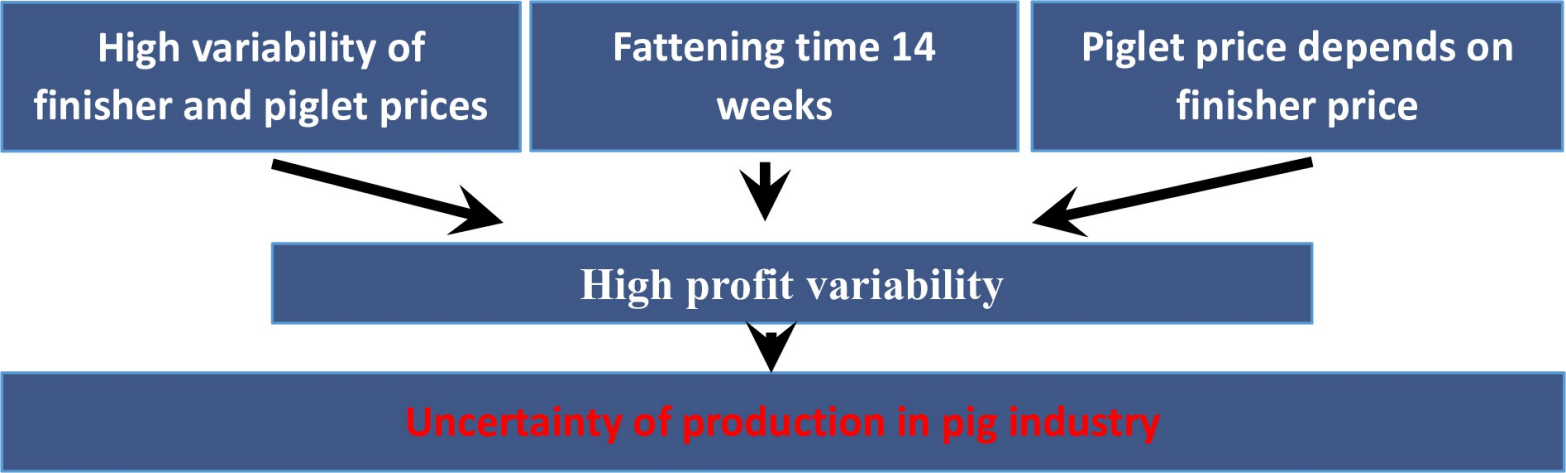
Current situation in the pig production sector. Source: Own elaboration.

To summarize, the current situation is one of considerable fluctuations in the prices of finishers and piglets, with a strong correlation between them. When adding to this the conditions of doing business (including a fattening period of 14 weeks and settlement between the two types of producers in this interval), we get a condition of large fluctuations in profit. This, in turn, makes pig production very precarious (Fig 6). A solution is therefore needed to increase the stability of the financial results [1–5,60].

An example of the application of the Algorithm, as presented in the section Algorithm as an innovative method—assumptions and their rationale, is shown in S1 Fig, which provides calculations for weeks 12 to 30 of 2022. Assuming that fattening starts in week 16, the sale of finishers would occur in week 30. Week 30 is the reference week for the explanations to the table. As an example, to calculate the profit of a finisher producer according to the current situation for week 30, the revenue of week 30, the price of piglets 14 weeks earlier (week 16), and the average costs of the preceding 14 weeks (week 16 to week 29) are used. Under market conditions, the finisher producer would make a profit of EUR 5.33 per unit, while the piglet producer would record a loss of EUR 3.63 per unit. By applying the Algorithm, the surplus across the chain (EUR 1.70 per unit) is divided according to the 2017–2022 proportions between the two producers. It is noteworthy that both the finisher producer and the piglet producer achieved a positive financial result—approximately EUR 0.75 per unit and EUR 0.95 per unit, respectively. As shown in Chart 4 in the next subsection, there were also periods between 2017 and 2022 when it would have been the finisher producer that would have avoided a loss by applying the Algorithm.

### Evaluation of the algorithm’s performance

**Fig 7:**
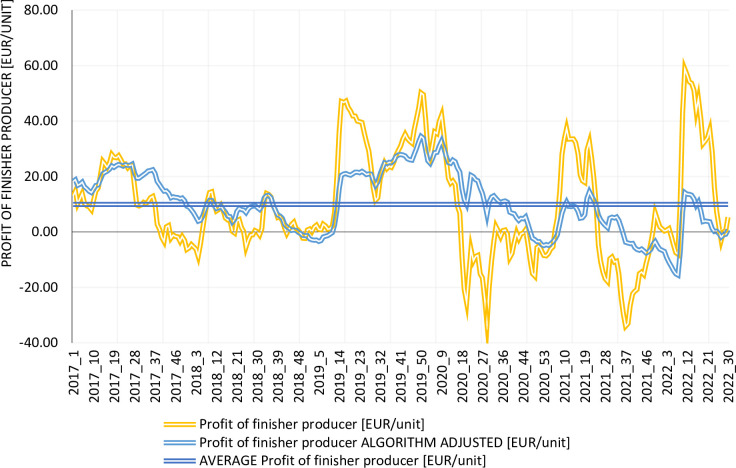
Finisher producer profit by current situation and algorithm, 2017–2022. Source: Own elaboration. Note: The average profit of the finisher producer is the same in the current situation as with the Algorithm, so the graph presents one line for the average profit in both situations.

As shown in Fig 7, implementing the Algorithm significantly reduces the profit fluctuations of the finisher producer. Furthermore, in most periods where a negative financial result was achieved, it is now positive, or the level of loss is much lower. Importantly, the average profit remains the same.

**Fig 8:**
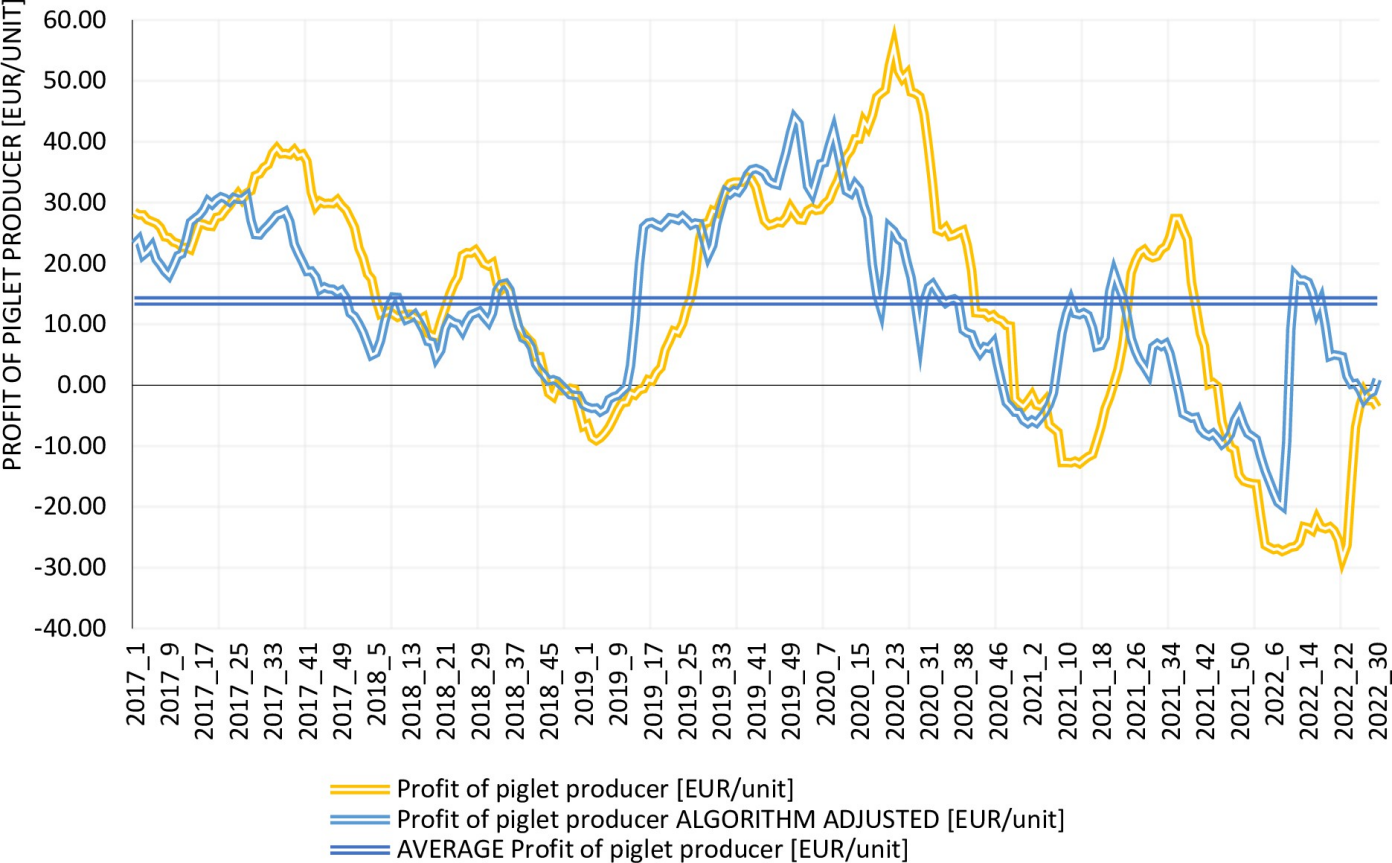
Piglet producer profit by current situation and algorithm, 2017–2022. Source: Own elaboration. Note: The average profit of the piglet producer is the same in the current situation as with the Algorithm, so the graph presents one line for the average profit in both situations.

Similar conclusions can be drawn when comparing the profit of the piglet producer before and after implementing the Algorithm (Fig 8). This means that more stable results are achieved and that the uncertainty associated with profit decreases. According to Ménard [35] reducing uncertainty results in lower transaction costs, and this has a positive impact on the development of the sector. Palát [55] argues that increasing production profitability is only possible by improving internal farm conditions (primarily cost efficiency). We do not disagree with this statement but we also show that at least the important problem of profit variability can be relatively easily solved. More stable profit enables to make short- and medium-term planning more efficient.

Martins [21] argue that only by achieving as large a difference as possible between the production costs (produce cheaply) and the revenue from the sale of piglets or finishers (sell at as high a price as possible) can one increase the sustainability of the pig livestock sector, improve the financial result, and reduce the risk of loss. However, reducing costs is increasingly difficult and, for some farms, is out of reach due to the excessive cost of investing in solutions to reduce operating costs.

In this context, the Algorithm at least makes it feasible to reduce profit fluctuations and avoid losses (even if the average profit remains the same) through a simple procedure of shifting the settlement date between the producers of finishers and piglets. This settlement consists of distributing the surplus once the finishers are sold. This surplus is, in most cases, positive so that both producers achieve a financial result above 0. In addition, the Algorithm is cost-free for both types of producers (ignoring the transaction costs of entering into a contract). The application of the Algorithm can therefore be regarded as an improvement in the Pareto sense—an improvement in the situation of the finisher producer occurs without a deterioration in the situation of the pig producer in the long term.

**Fig 9:**
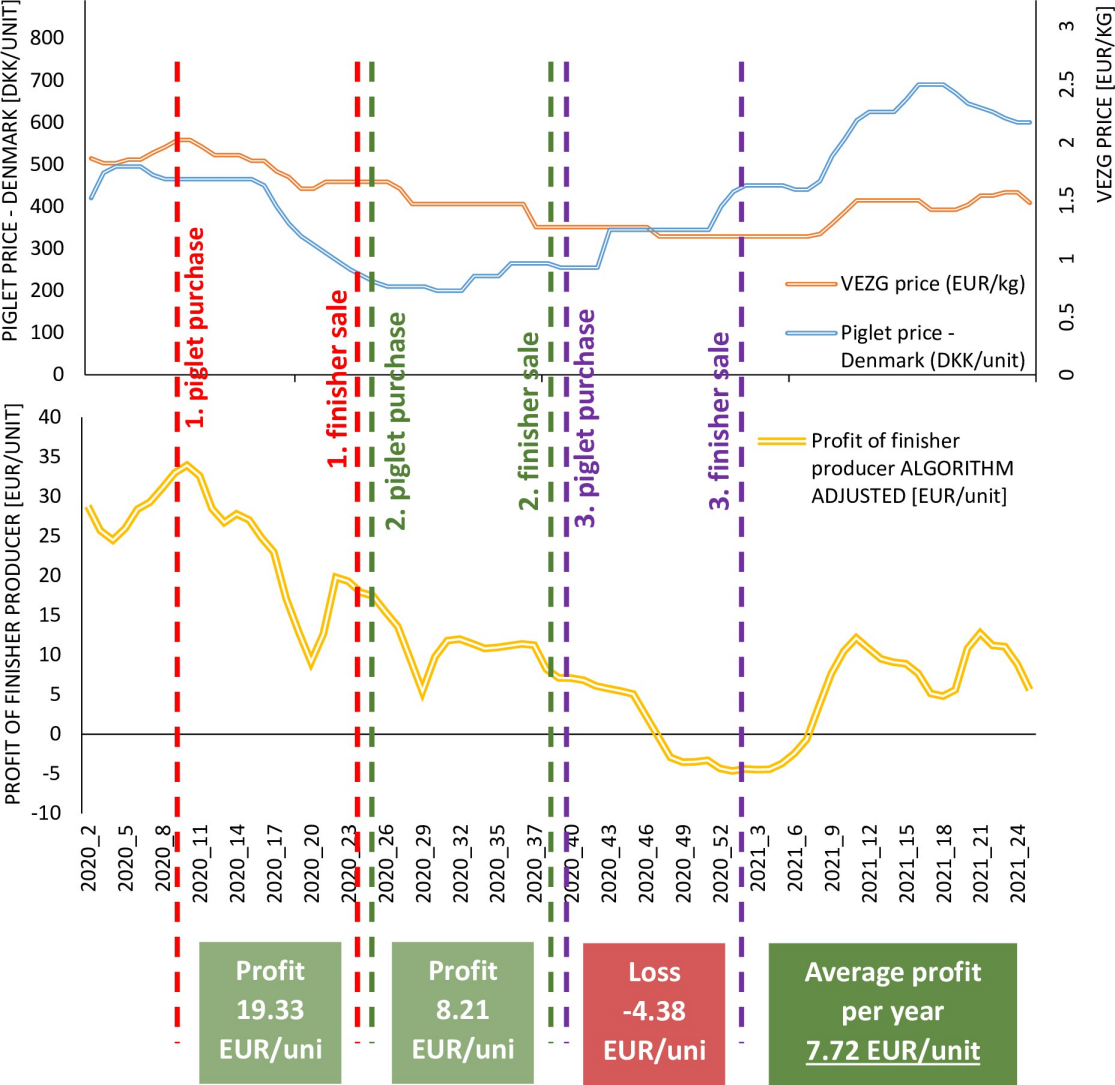
Profit from finisher sales in 2020 –algorithm adjusted. Source: Own elaboration.

To show the impact of the Algorithm on the sustainability and long-term profitability of finisher production, Fig 9 shows the financial result for the same period as in Fig 5. With the Algorithm, there is a positive average financial result (almost EUR 8/unit) during the year, whereas under current conditions there is an average loss in 2020 of just over EUR 7.5/unit.

As demonstrated in Table 4, introducing the Algorithm results in a significant reduction in profit fluctuations for both the finisher and piglet producers. The coefficient of variation for the situation according to the current conditions is significantly higher than for settlements that use the Algorithm. For the entire time range, 2017–2022, the standard deviation of the profit drops from 19.28 to 10.62 EUR/unit (by 45%) and from 20.17 to 14.13 EUR/unit (by 30%) for the finisher and piglet producers, respectively. Similar conclusions can be drawn from the analysis of individual years. The standard deviation for the Algorithm is in most cases lower than for the current situation. This means that introducing the Algorithm reduces the uncertainty of doing business in the pig production sector. This is particularly important, especially given the strong fluctuations in average profit from year to year. In addition, shorter periods of negative profit (or eliminating them altogether) mean that producers do not need to take out loans to sustain operations. This also reduces operating costs.

**Table 4:** Descriptive statistics for the financial result, 2017–2022.

Year	Profit generation method	Average profit [EUR/unit]		Share in profit		Standard deviation of profit	
		*Finisher producer*	*Piglet producer*	*Finisher producer*	*Piglet producer*	*Finisher producer*	*Piglet producer*
2017–2022	Market	10.31	13.72	44%	56%	19.28	20.17
	Algorithm					10.62	14.13
2021*	Market	1.30	2.52	34%	66%	8.66	6.27
	Algorithm					2.18	4.23
2020	Market	3.86	28.83	11%	89%	9.26	12.97
	Algorithm					1.93	14,42
2019	Market	25.57	14.20	64%	36%	11.51	8.38
	Algorithm					12.02	6.67
2018	Market	3.74	11.28	25%	75%	2.88	5.19
	Algorithm					1.68	5.08
2017	Market	10.47	30.35	25%	75%	5.76	11.53
	Algorithm					4.08	11.84

Notes

*2022 was not considered separately (like each year from the range 2017–2021) as the values do not reflect full cycle for the year.

Source: Own elaboration.

**Fig 10:**
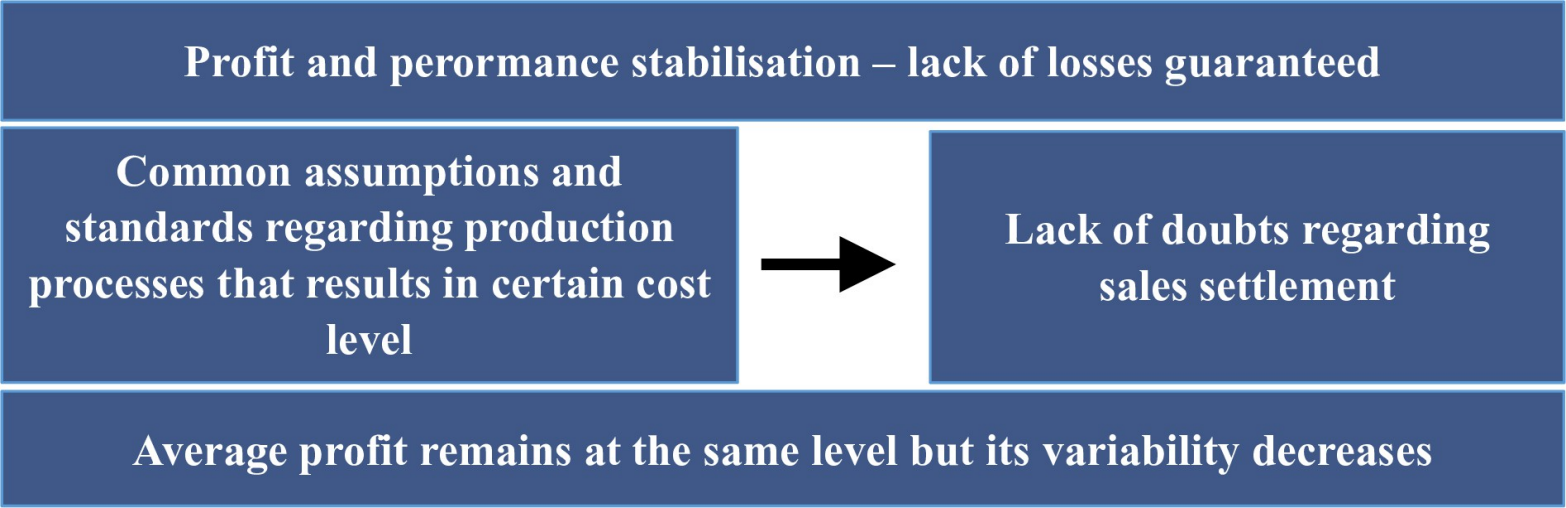
Benefits of the algorithm. Source: Own elaboration.

To summarize, introducing the Algorithm contributes to stabilizing profits, thereby reducing the uncertainty of the pig business (Fig 10) and, consequently, transaction costs [35]. Importantly, the average profit remains the same, but its fluctuations decrease. As highlighted by many authors [3,5,21,23,70,71] several studies have shown that strong fluctuations in the financial outcomes of piglet and finisher producers are a major cause of instability in pig production.

Using the Algorithm seems particularly important for semi-subsistence farms, which are particularly sensitive to fluctuations in profit and have great difficulty in achieving long-term profitability and production stability. Importantly, this type of farm dominates the Polish production structure, making it collectively very influential on the levels and changes in pork livestock supply [17].

The Algorithm also appears to be an excellent alternative to contract fattening. As already mentioned, this form of cooperation with pork livestock recipients, to which low-subsistence farms that have problems with the instability of financial results are condemned, often results in breaking off cooperation with existing (usually domestic) piglet suppliers, which destabilizes the situation in the pig production sector. Importantly, smaller fattening piggeries can strike the right balance between the length of the chain and the quality of the product supplied, unlike in large corporations [19,20]. The Algorithm presented here allows smaller farms to stay in an open cycle model.

The entire solution is based on collaboration between the producers of finishers and piglets, which is a prerequisite for engaging with the Algorithm. However, as already mentioned, limited rationality related to cognitive limitations, agent opportunism, low social trust, and perceptual limitations may significantly affect the willingness to participate in solutions that require cooperation [43–45].

In this context, at least two objections may arise among potential users of the Algorithm. The first is postponing the settlement of the purchase of piglets until the sale of the finishers. This may raise doubts about the fairness of the distribution of the financial results. The second is the unequal proportions with which the profit is distributed to the two producers. This may be opposed by the finisher producer, who receives a smaller share of the surplus. These concerns are the main manifestations of cognitive and perceptual limitations about the Algorithm. However, they can be mitigated by increasing human and social capital, including trust [47].

Therefore, the Algorithm should always be presented in terms of the benefits of stabilizing financial outcomes in the long term and the importance of building networks of trust between the farmers. This can be done through workshop meetings with groups of farmers. These meetings should serve to network the participants of the pig sector involved in the Algorithm and help address their doubts. In particular, with regard to the first doubt indicated above, it should be communicated that the Algorithm makes assumptions and standards regarding receipts and costs, which are not subject to manipulation. Adherence to these assumptions guarantees the transparency of the after-sales settlement. In addition, the unequal distribution of the surplus is based on the desire to compensate the piglet producer for the greater risks involved in running the business. Moreover, this distribution of surpluses is based on historical data on the average shares of both types of producers in the chain’s profit.

## Conclusion

The pork livestock sector has faced unstable financial results for a long time, which affects the structure of producers. Some withdraw from production, while others sign agreements under contract fattening, which affects the ability to provide an adequate supply in terms of both quantity and quality. The issue of unstable financial results is aggravated by recent challenges, including the COVID-19 pandemic and the ASF epidemic.

Therefore, as part of our study, we proposed and evaluated a solution that guarantees the stability of financial results for pork livestock producers. Effective solutions of this type have thus far been lacking in the literature. The proposed tool, thanks to postponing the settlement of the sale of piglets to the day when the finishers are sold, eliminates the impact of fluctuations in the prices of finishers and piglets on the financial result of producers. This helps both types of producers to stabilize profit and minimize number of periods with a loss. An analysis of historical data from January 2017 to July 2022 showed that the above-described shift in the settlement of piglet purchases ensured that the profits of the producers of piglets and finishers remained at the same average level while dropping below 0 much less frequently than at present. Profit variability is also reduced, guaranteeing operational stability. If the Algorithm had been implemented between 2017 and 2022, the profit variability of the piglet producer would have fallen by around 45%.

Ensuring a positive economic outcome for fattening farms allows for an alternative to contract (hotel) fattening. This allows producers to maintain their autonomy, which is especially true for semi-subsistence farms, which have more opportunities to realize the idea of a short supply chain. At the same time, finisher producers, while gaining a guarantee of profit stability, have a direct impact on piglet producers. They give them a warranty for the sale of piglets, encouraging them to stay in business. Importantly, linking the producers of finishers and piglets, both by settlement and by piglet sale and purchase agreements, is an important tool for sustainable vertical integration in the pig sector.

To date, EU agricultural policy has emphasized the role of integration in the sector, but direct support for producer groups has mainly come down to subsidies for the operation of agricultural producer groups during the first five years of operation. It seems that a sustainable improvement in the situation of pork livestock producers would require solutions dedicated to this group of producers. In this context, it would be appropriate to introduce subsidies for producers of piglets and finishers, and they would sign a contract for settlement according to the Algorithm. Such subsidies could be a form of remuneration for farmers for the transaction costs incurred.

Among the limitations of our study, it can be stated that an evaluation of the proposed Algorithm has been carried out for Poland, which is the example of country with relatively low level of social capital, especially when it comes to farmers population. In such conditions we need to look for solutions that do not require full integrations (such as cooperatives) which are desired but seem to be not possible yet. The proposed algorithm can be, however, treated as a step to more advanced future arrangements as it promotes building social trust. Sharing knowledge and communicating the transparency of the solution decrease the fear of using Algorithm. Another limitation is related to the fact that some costs (such as veterinary or electricity costs) are assumed here to be fixed. In future research the specific methods to keep these costs up to date should be introduced.

The phenomenon of the pig cycle, which is the direct incentive for the preparation of the Algorithm, is a universal phenomenon, and it also occurs in other local markets. An evaluation of the effectiveness of the Algorithm in local markets with a higher degree of integration could provide a fruitful line for further research. Promising results could come from considering both the effectiveness of changing the form of integration from those currently used to one based on the Algorithm and on farmers’ willingness to be involved in such solutions. Concerning the latter, experiments could be carried out to identify the factors that affect the eagerness of producers to sign an agreement within the Algorithm framework.

## Supporting information

S1 Fig Example of algorithm application for fattening starting in a week 16. of 2022. Source: Own elaboration.
(TIF)

S1 Table Studies of the profitability and efficiency of pork production. Source: Own elaboration.
(DOCX)

S1 File Detailed assumptions for calculation of cost of production. (DOCX)
