# Supplementary figures and images for: A new financial settlement approach to stabilize profitability of pig production
Source: PLoS One. 2024 Jun 10;19(6):e0304949. doi: 10.1371/journal.pone.0304949 (PMC11164379; doi:10.1371/journal.pone.0304949)

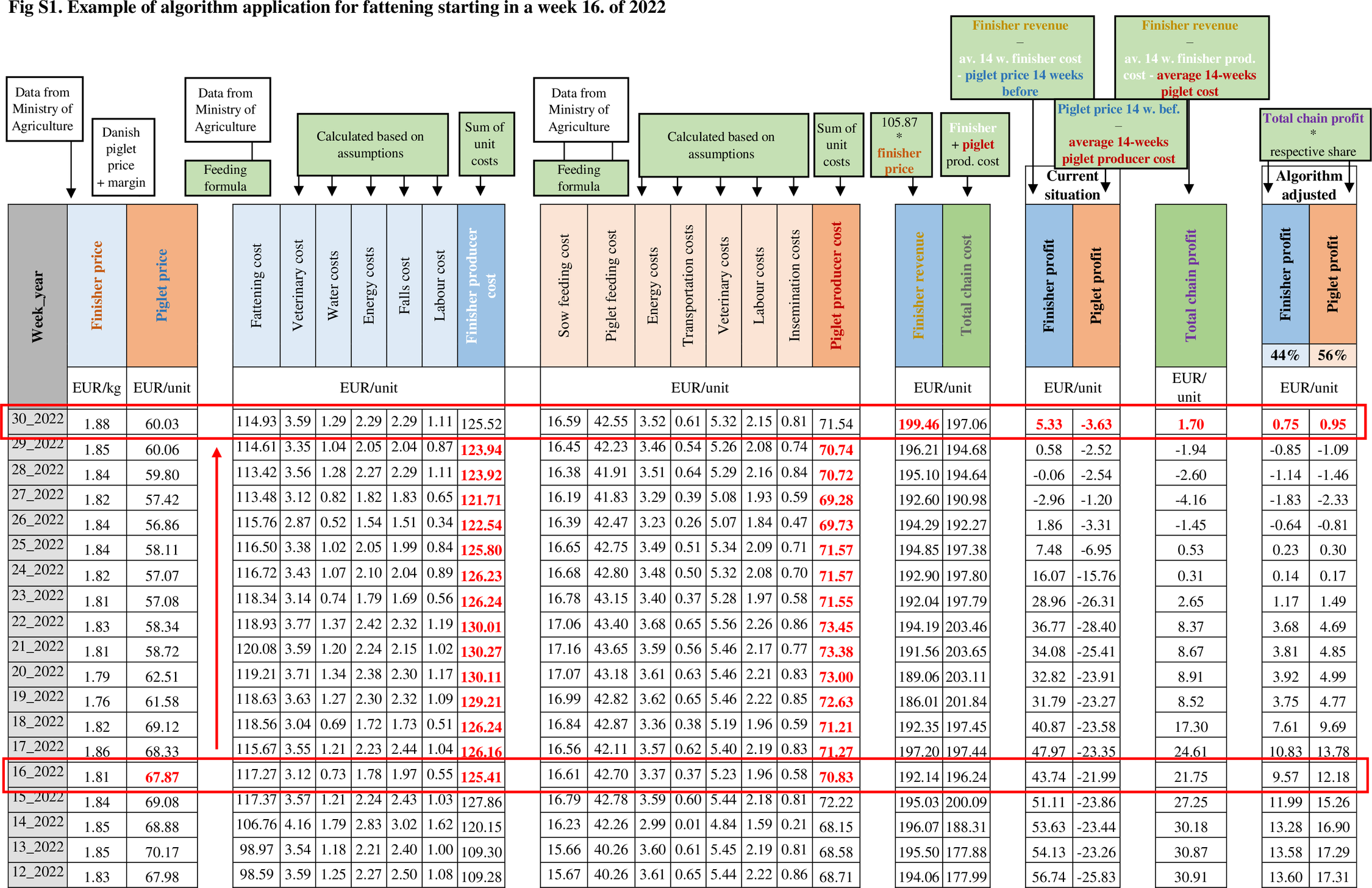

Supplement: S1 Fig — Source: Own elaboration. (TIF) [file pone.0304949.s001.tif]
