# Supplementary material for: A new financial settlement approach to stabilize profitability of pig production
Source: PLoS One. 2024 Jun 10;19(6):e0304949. doi: 10.1371/journal.pone.0304949 (PMC11164379; doi:10.1371/journal.pone.0304949)
Supplement: S1 Table — Source: Own elaboration. (DOCX) [file pone.0304949.s002.docx]

**Table S1. Studies of the profitability and efficiency of pork production**

| **Author (s) and Year** | **Country/**  **region** | **Research problem or purpose** | **Main results** |
| --- | --- | --- | --- |
| Sharma et al., 1999 | Hawaii | The potential of the pig industry based on determining the operational efficiency of commercial pig farmers. | Significant inefficiencies in pig production demonstrated. |
| Rowland et al., 1998 | Kansas, Central U.S.A. | The economic competitiveness of the farrow-to-finish operation was examined by estimating the relative productivity of the company. | Efficient farms feature significant pig production, generate a large proportion of their income from pig production, and have a lower debt-to-asset ratio. |
| Lansink, Reinhard, 2004 | Dutch | Improving the technical, economic, and environmental performance of Dutch pig farms. | The average technical efficiency of the farms is higher than with currently available technologies. |
| Galanopoulos et al., 2006 | Greece | Degree of technical efficiency and scale of commercial pig rearing. | The average technical efficiency in the DEA model was 0.83, indicating that there is great potential for more efficient use of inputs. |
| Petrovska, 2011 | Republic of Macedonia | Level of technical efficiency of pig farms in the Republic of Macedonia. | Technical efficiency analyzed in terms of constant returns to scale is always lower, with an average of 75%. According to variable returns to scale, the average technical efficiency from an input perspective is 90%, and from an output perspective, 87%. |
| Adetunji, Adeyemo, 2012 | Oyo State, Nigeria | Economic efficiency of pork production. | Pig farmers were generally economically efficient, able to achieve greater improvements through easy access to institutional credit, expansion of pens, better breeding stock, and provision of technical assistance. The cost of feed and labor had a significant impact on production volumes. |
| Latruffe et al., 2013 | Hungary | The impact on production and technical efficiency, if environmental regulations were fully implemented, was examined. | Pollution can be reduced without affecting production levels, and pig farmers have an incentive to reduce nitrogen pollution to increase productivity even in the absence of regulation. |
| Labajova et al., 2016 | Sweden | The impact of farm management practices on sustainability and overall efficiency. | Advisory bodies and policy makers should develop distinct approaches depending on which input and output efficiencies they intend to improve, as well as the type of pig production. |
| Ly et. al., 2016 | Vietnam | Technical efficiency in domestic pig production has been investigated and attempts have been made to determine what factors influence it. | The overall technical efficiency (TEcrs) was 80.40%. Factors affecting TEcrs include liveweight of the fattening pig, breeding time, experience and education of family members for pig production, income from pigs, access to credit, and veterinary services. |
| Nadal-Roig et al., 2020 | Brak danych | Addressing tactical decisions on production planning, increasing flexibility, improving coordination and overall pig production under uncertainty of future selling price. | The stochastic solution for the case study provides an optimal first-stage production plan for purchasing 1016 piglets per week in addition to the 775 already produced outside of the rental rearing and fattening farms, taking into account various scenarios. The model can identify inefficiencies and bottlenecks in the system. |
| Martins et al., 2021 | Brazil | How technical and management support from buyers affects the productivity and investment capacity of Brazilian pig farmers. | The relationship between buyer support and investment capacity was significant only in the finisher sample. The investment capacity of farmers positively influences the performance of piglet breeders. For finishers, investment capacity affects only financial performance. |
| Wang et al., 2021 | China | Applying stochastic frontier analysis to calculate the cost-effectiveness of fattening pigs, sows, and piglets in the three stages of pig production and focus on the impact of environmental regulations on cost-effectiveness. | The cost-effectiveness of fattening pigs, sows and piglets in China was 0.77, 0.79, and 0.53, respectively, and yield losses were 23%, 21%, and 47%, respectively. |
| Boyle et al., 2022 | Ireland | Pork production in Ireland has undergone huge changes over the past 60 years, from pigs raised mainly on dairy farms as a side production stream to an industry with one of the highest average herd sizes in Europe. | The pig industry worldwide faces a huge challenge to meet the protein demands of a growing global population, while improving pig health and welfare, minimizing environmental damage, and maintaining economic sustainability. |
| Raja et al, 2022 | Tamil Nadu | Profitability in pig farming was investigated and an analysis was made of the various factors influencing pig production and their productivity levels. | Large pig farms had a minimum production cost and maximum net return per farm per year. The overall benefit-cost ratio was 1.46, revealing the profitable nature of pig farms. |
