# Supplementary material for: A new financial settlement approach to stabilize profitability of pig production
Source: PLoS One. 2024 Jun 10;19(6):e0304949. doi: 10.1371/journal.pone.0304949 (PMC11164379; doi:10.1371/journal.pone.0304949)
Supplement: S1 File — (DOCX) [file pone.0304949.s003.docx]

**Table A1. Data sources**

| **Variable** | **Frequency** | **Data source and original unit** | |
| --- | --- | --- | --- |
| **Finisher price** | *weekly* | Polish Ministry of Agriculture [PLN/kg] | <https://www.gov.pl/web/rolnictwo/rynek-wieprzowiny> |
| **Piglet price** | *weekly* | Danish exchange [DKK/piece]; price for PRRS negative  + trader's margin of EUR 13,66/unit. recalculated to PLN. | <https://www.cenyrolnicze.pl/dania-spf> |
| **Currency exchange rate EUR/DKK/PLN** | *daily* | National Bank of Poland (central bank): <https://www.cenyrolnicze.pl/kursy-walut> | |
| **Rapeseed meal prices** | *daily* | Price on MATIF stock exchange [PLN/t] | <https://www.agrolok.pl/notowania/notowania-sruty-rzepakowej.htm> |
| **Soybean meal prices** | *daily* | Price in the port of Gdynia [PLN/t] | <https://www.agrolok.pl/notowania/notowania-sruty-sojowej.htm> |
| **Other crops prices** | *monthly* | Polish Ministry of Agriculture [PLN/t] | <https://www.gov.pl/web/rolnictwo/notowania-w-2021-r7> |
| **Treatment costs for fattening pigs** | *constant* | PLN 16/unit | The costs were determined on the basis of a reference methodological publication by the Danish institution SEGES (2022) and a manual on pig farming (Pawłowski, 2020). In addition, the assumptions were validated on the basis of the experience of Polish pig producers. |
| **Utility costs (water, electricity) in finisher production** | *constant* | PLN 14.8/unit |  |
| **Labour costs – finisher** | *constant* | PLN 4.17/unit | Determined on the basis of the assumptions indicated in Table A5 in the Appendix. |
| **Costs of falls in finisher production** | *weekly* | Variable costs, determined on the basis of the price of the piglet and the formulas shown in Table A7 in the Appendix. | |
| **Treatment costs for piglets and sows** | *constant* | PLN 25/unit | The costs were determined on the basis of a reference methodological publication by the Danish institution SEGES (2022) and a manual on pig farming (Pawłowski, 2020). In addition, the assumptions were validated on the basis of the experience of Polish pig producers. |
| **Utility costs (water, electricity) in piglet production** | *constant* | PLN 16.4/unit |  |
| **Labour costs – piglet** | *constant* | PLN 9.87/unit | Determined on the basis of the assumptions indicated in Table A4 in the Appendix. |
| **Piglet transportation costs** | *weekly* | PLN 2.5/unit | Determined on the basis of the assumptions indicated in Table A6 in the Appendix. |

**Table A2. Labor costs in piglet production – assumptions**

| **Category** | **Assumption** | **Unit** |
| --- | --- | --- |
| Sow number | 200 | - |
| Labor time | 8,35 | h/year |
| Hourly wage rate | 25 | PLN/h |

Source: own elaboration.

**Table A3. Labor costs in finisher production – assumptions**

| **Category** | **Assumption** | **Unit** |
| --- | --- | --- |
| Labor time | 10,00 | min/unit |
| Hourly wage rate | 25 | PLN/h |

Source: own elaboration.

**Table A4. Piglet transportation costs – assumptions**

| **Category** | **Assumption** |
| --- | --- |
| Piglet number | 400 |
| Distance (km) | 100 |
| Rate per km (PLN) | 5 |
| Transportation calculated in both directions | |

Source: own elaboration.

**Table A5. Assumptions for calculation of falls costs (finisher)**

| **Weight of a fallen porker** | **Number of fallen animals by weight** | **Cost of falls per unit** | **Total cost of fall** |
| --- | --- | --- | --- |
| X < 50 | 3 | piglet price +(20*3*1,05) | No. of animals * cost of falls per unit |
| 50 < X < 80 | 3 | piglet price +(50*3*1,05) |  |
| 80 < X < 100 | 10 | piglet price +(20*3*1,05) |  |
| 100 < X < 130 | 3 | piglet price +(70*3*1,05) |  |

**Table A6. Feeding schedule for finishers, sow and piglets (in kg per cycle)**

|  | **Finisher** | **Low-pregnancy sow** | **High-pregnancy sow** | **Piglet** |
| --- | --- | --- | --- | --- |
| Soybean/soybean meal | 17.65 | 10.00 | 14.15 | 9.94 |
| Rapeseed / rapeseed meal | 10.10 | 14.99 | - | - |
| Soybean oil | 1.54 | - | 1.60 | 3.06 |
| Oats |  | 51.65 | 7.07 | - |
| Bran |  | 23.32 | 4.72 | - |
| Triticale | 68.88 | 58.31 | - | - |
| Barley | 88.13 | 99.96 | 31.13 | 27.88 |
| Wheat | 65.89 | 66.64 | 31.88 | 29.08 |
| Premiks | 7.71 | 8.33 | 3.77 | 2.91 |
| Probiotic | 0.85 |  |  |  |
| Acidifier | 0.28 |  |  |  |
| Supplements | 0.68 |  |  |  |
